# Ultraviolet Spectrophotometric Method for Determination of Gelatin Crosslinking in the Presence of Amino Groups

**DOI:** 10.4103/0975-1483.62223

**Published:** 2010

**Authors:** RN Kale, AN Bajaj

**Affiliations:** *Department of Pharm. Sciences, C.U. Shah College of Pharmacy, SNDT Women’s University, Juhu Tara Road, Santacruz (W), Mumbai - 49, India*

**Keywords:** Chromotropic acid reagent, colorimetric assay, gentamicin sulfate, trinitrobenzene sulfonic acid, ε-amino groups

## Abstract

The study was carried out to develop procedure for determining concentration of formaldehyde to be used for crosslinking of gelatin in the presence of drugs having amino groups. Gentamicin sulfate was used as a drug candidate due to its high content of amino acids. Gelatin crosslinking is accelerated by aldehyde-containing compounds and inhibited by amino group-containing compounds. The major modifications from already existing procedures are that the trinitrobenzenesulphonic acid (TNBS) reaction is used to detect e-amino groups of Type A gelatin in the presence of formaldehyde and further it is supported with colorimetric analysis of free formaldehyde content using a chromotropic acid regent. Since formaldehyde crosslinks amino groups, the TNBS assay can be effectively utilized for determination of complete crosslinking of gelatin with analysis of free amino acid content in crosslinked formulation. The effect of the presence of amino groups on gelatin crosslinking was estimated in the presence of gentamicin sulfate. The ε-amino content of uncrosslinked Type A gelatin was found to be 28.6 mol/gelatin molecule of 1000 residues and in case of crosslinked gelatin it varies with varying concentration of formaldehyde. The procedure stated here should be applicable to a broad range of drugs containing amino groups which are used along with gelatin or other proteinaceous materials which are applicable after crosslinking with formaldehyde.

## INTRODUCTION

Structures containing proteins such as albumin, collagen, and gelatin have been investigated as biomaterials for drug delivery.[1] Among all these materials, gelatin is the most suitable biomaterial candidate since it is derived from collagen which is main component of human body. Its physicochemical properties can be suitably modulated due to existence of many functional groups as amino groups and it has not shown any antigenicity.[2,3]

One of the important modification of any polymer for drug delivery or as biomaterial is crosslinking between amino groups.[3] A convenient assay of ε-amino groups could be used to determine the number of lysine residues during crosslinking. Lysine is an essential amino acid and has positively charged ε-amino groups (a primary amine) which are crosslinked while chemical modification.

Gelatin can be crosslinked physically by thermal heating, ultraviolet irradiation[4] and chemically by several crosslinking agents such as formaldehyde,[5] glutaraldehyde,[6] water soluble carbodiimide, diepoxy compounds, diisocyanates, and dextran aldehydes. Formaldehyde is most common and economical crosslinking agent used for gelatin crosslinking.[7]

Gelatin crosslinking is accelerated by formaldehyde, glutaral dehydes, glyceraldehydes, hydrogen peroxide, benzene, sulphonic acid, and guanidine hydrochloride and is inhibited by amino groups such as glycine, lysine, phenyalanine, and glycerin. Drug with similar functional groups were expected to show parallel behavior.[8] William, Bubnis, and Ofner III have introduced modified procedure for determination of amino groups in proteinaceous materials. Further variation and modification of this procedure are used to study the effect of additional amino groups in the form of drug on the crosslinking of proteinaceous material.

**Figure 1:**
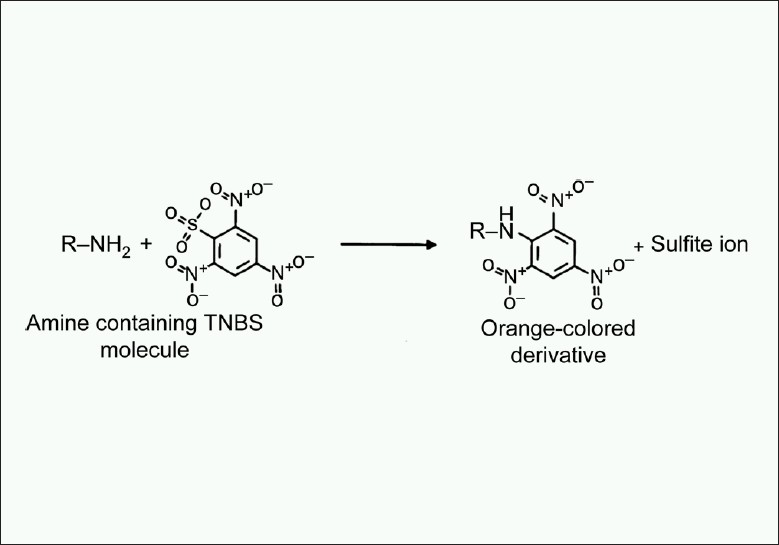
Reaction of trinitrobenzenesulphonic acid with a primary amine. containing molecule to produce trinitrophenyl derivative and sulfite ion

Earliest procedures did not differentiate between α and ε-amino groups. Some procedures measured orange color absorbance at 420 nm but were required to account for an interfering complex formed between the trinitrophenyl (TNP) derivative and the sulfite ion by-product [Figure 1].

**Figure 2:**
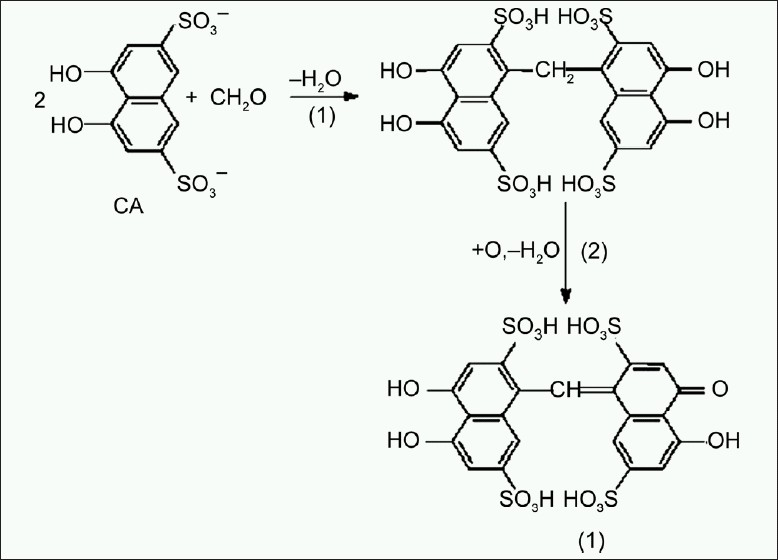
Two step reaction of development of purple colored chromogenic derivative. Chromotropic acid (I): Chromogenic derivative

Kakade and Liemen introduced an extraction step which removed both excess unreacted TNBS and TNP-α-amino derivatives. The reagent trinitrobenzene sulfonic acid (TNBS) has been used as a UV chromophore to determine ε-amino groups at 346 nm. TNBS reacts specifically with ε-amino groups and formaldehyde also crosslinks ε-amino groups. This concept can be utilized for determination of free primary amino groups for confirmation of complete crosslinking of gelatin molecules.[9,10] Chromotropic acid [1,8-dihydroxynaphthanene-3,6-disulphonic acid] reagent was used for estimation of excess formaldehyde colorimetrically. Formaldehyde develops characteristic purple color with chromotropic acid reagent [Figure 2] that shows maximum absorbance at 570 nm.[11]

Since gentamicin sulfate has high concentration of primary amino groups which can be responsible for inhibition of gelatin crosslinking with formaldehyde, it is used as drug candidate for illustration of this analytical method.

The results of this study highlight the method for determination of formaldehyde to be used in formulations containing gentamicin sulfate. The method mentioned here will be also helpful in deciding the concentration of formaldehyde to be used for crosslinking of gelatin in the presence of other drugs having free amino groups in its chemical structure. Colorimetric estimation of excess formaldehyde using chromotropic acid is introduced to assure complete crosslinking of gelatin with safe concentration of formaldehyde.

## MATERIALS AND METHODS

### Reagents

Type A gelatin prepared by acidic treatment of porcine skin was procured from Nitta Gelatin [Canada]. Porcine gelatin powder was used without further purification. The moisture content of powder during storage was approximately 9.0%. The pH of the 5% solution was 4.8. Formaldehyde solution [37-41%], conc. sulfuric acid, sodium bicarbonate, hydrochloric acid, and chromotropic acid were ordered from S.D. Fine chemicals, Mumbai. Chromotropic acid reagent consisted of 50 mg of chromotropic acid per 100 ml of mixture of 9 ml concentrated sulfuric acid and 4 ml of water. TNBS [≥98% pure] was purchased from Sigma Aldrich. Gentamicin sulfate was procured from Yantai Justaware Pharmaceuticals Co. Ltd, China. The water used was distilled water. All the chemicals used were ACS reagent grade.

### Apparatus

An UV-VIS spectrophotometer (Analytikjena, SPECORD 205) was used for absorbance measurement.

#### Trinitrobenzenesulphonic acid assay procedure

Approximately 11 mg of solid sample or 1-2 ml of dissolved protein (3.85 ± 0.200 mg/ml) was placed in a 50 ml screw cap test tube. One milliliter of 4% NaHCO_3_(pH 8.8) and 1.00 ml of 0.50% TNBS were added to the tubes. The reaction mixture was heated at 40°C for 4 h with mild shaking. Three milliliter of 6 N HCl was added and mixture was autoclaved at 120°C and 15-17 psi for 1 h to hydrolyze and dissolve any insoluble matter. The hydrolysate was then diluted to 5.0 ml with water. The hydrolysate dilution was extracted with three 20 ml portions of ethyl ether to remove excess unreacted TNBS. A 5.0 ml aliquot of the aqueous phase was removed and heated for 15 min in hot water bath to evaporate residual ether. The aliquot was diluted with 15.0 ml of water and the absorbance was measured at 346 nm in the Jasco double-beam spectrophotometer.[10 –12]

All samples were read against reagent blank which was prepared with same procedure. However, the HCl was added before the addition of TNBS to prohibit any reaction of TNBS with protein. Trials containing four to five replicates were repeated three to four times.

The ε-amino determination was conducted on uncrosslinked gelatin. The effect of formaldehyde on concentration of ε-amino groups was determined and influence of gentamicin sulfate was checked further by determination of ε-amino groups by following Eq. 1.

1 $$\frac{\mathrm{Moles}\;\mathrm{of}\;\mathrm{lys}}{\mathrm{Moles}\;\mathrm{protein}}\;=\;\frac{2\left(\mathrm{Absorbance}\right)\left(0.020\;1\right)\;\left(\mathrm{MW}\right)}{\left(1.46\;\times \;10^{4}\;1/\mathrm{mol}\;\mathrm{cm}\right)\left(b\right)\left(x\right)}\;$$

Where MW is the protein molecular weight with the units of g/mol, 1.46 l/mole cm is the molar absorptivity of TNP-lys, *b* is the cell path length in cm, and *x* is the sample weight in grams. Gelatin results were calculated by modifying Eq. 1. to express results as moles lys/g gelatin due to uncertainty of the molecular weight of gelatin.

#### Colorimetric estimation of free formaldehyde by Chromotropic acid reagent

Compositions of gelatin were further tested for residual formaldehyde content as procedure described below. About 0.5 ml of the solution was transferred to a 50 ml glass stoppered test tube and 10 ml of chromotropic acid reagent was added, the tube was stoppered and heated in a water bath for 30 min. The absorbance of the resulting solution was taken at 570 nm in SPECORD double-beam spectrophotometer.[13]

## RESULTS AND DISCUSSION

The determination of formaldehyde content in formaldehyde crosslinked formulations is essential for safe and effective use of the modified formulation. To evaluate concentration of formaldehyde for gelatin crosslinking in the presence of drug containing primary amino groups, gentamicin sulfate was directly incorporated in gelatin solution along with varying concentrations of formaldehyde. Gentamicin sulfate was included in the study due to its wide use in biomaterials for its antibacterial property and due to the presence of amino groups in its chemical structure, which interferes with gelatin crosslinking.[14,15]

**Figure 3:**
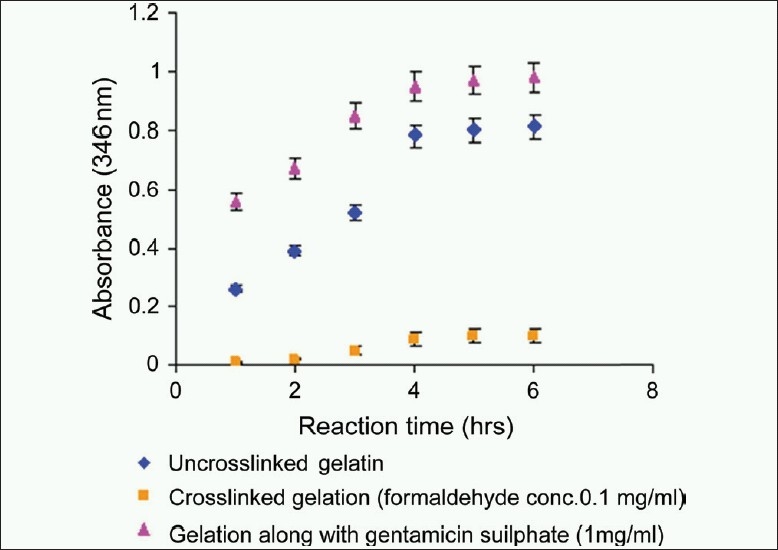
Determination of time for the completion of reaction of 2,4, 6-trinitrobenzenesulphonic acid with uncrosslinked gelatin, crosslinked gelatin, and gelatin along with gentamicin sulfate. Absorbance was measured at 346 nm against reagent blank. Each data point is the average of five replicates and bars represents percentage

TNBS reacts predominantly with primary amines. The duration of the complete reaction of TNBS was determined by absorbance measurements of uncrosslinked gelatin, crosslinked gelatin, and gelatin along with gentamicin sulfate after TNBS reaction time of 1 to 6 h. The result indicated that 4-5 h are required for completion of TNBS reaction [Figure 3]. Table 1 shows the results of ε-amino group content of uncrosslinked gelatin. Three trials yielded average 28.6 × 10^–5^mol/g gelatin. The value corresponds to 28.6 ε-amino groups on one gelatin molecule of 1000 amino acid residues. These values are not well established since amount of amino groups depends on source collagen for gelatin.

**Table 1:** Determination of ε-amino groups in type A uncrosslinked gelatin

Trial	*n*	ε-amino groups moles/g (×10^5^)	SD	Coefficient of variation
1	5	28.8	0.2	0.6
2	5	28.4	0.35	1.2
3	5	28.6	0.3	1
Average		28.6	0.4	1.3

Content of ε-amino groups were further determined for crosslinked gelatin with varying concentration of formaldehyde. This study is supported with determination of excess formaldehyde in the same formulation using chromotropic acid reagent. Formaldehyde condenses with chromotropic acid reagent in the hot sulfuric acid and is transferred to its *para*-quinoidal form, which produces typical purple color which has maximum absorption at 570 nm. Maximum color intensity was attained within 10 min of heating at 90°C used throughout the study.

**Figure 4:**
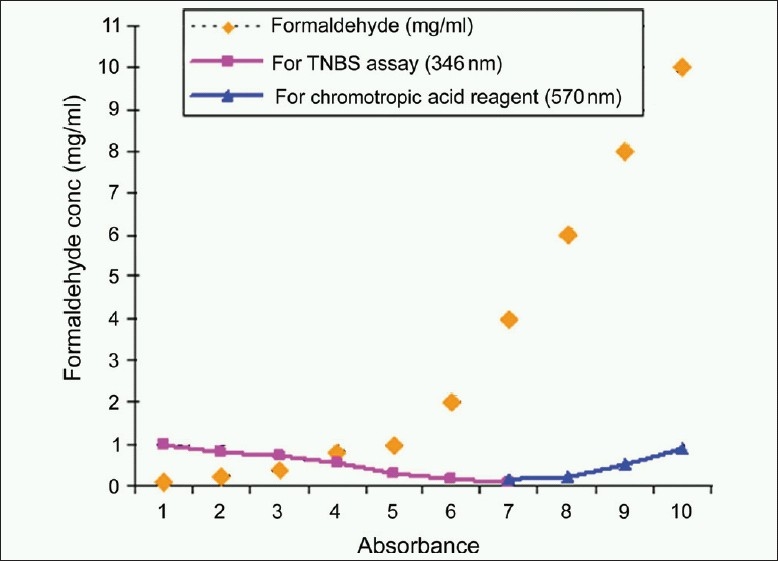
Study of gelatin crosslinking using the trinitrobenzenesulphonic acid assay and chromotropic acid reagent. As there is an increase in concentration of formaldehyde, the absorbance for the trinitrobenzenesulphonic acid assay decreases indicating reduction in moles of lysine in gelatin. After complete crosslinking of gelatin, increase in absorption at 570 nm indicates excess formaldehyde present in the formulation

Change in concentration of ε-amino groups of gelatin after crosslinking with formaldehyde in the range of 1-8 mg/ml and same along with gentamicin sulfate was determined. There is a decrease in concentration of ε-amino groups as the concentration of formaldehyde increases [Figure 4].

**Figure 5:**
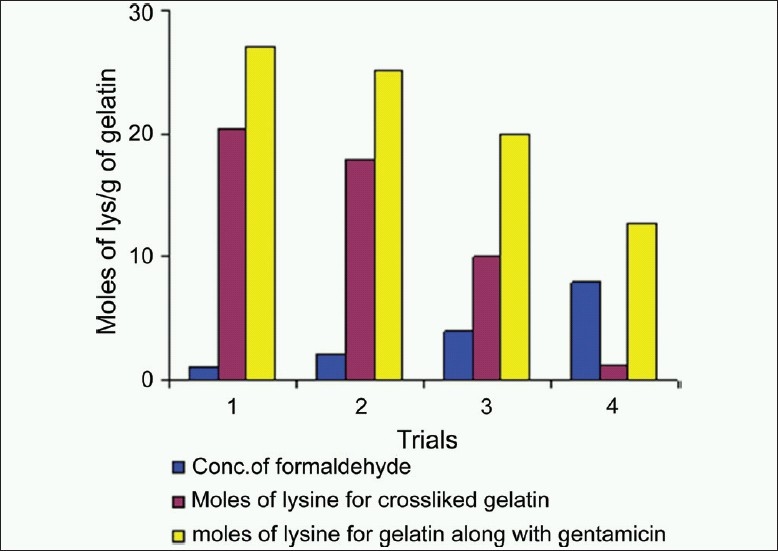
Comparative data of determination of moles of lysine per g of gelatin using trinitrobenzenesulphonic acid calculated using Eq.1. Each column is the average of five replicates. It shows increase in moles of lysine per g of gelatin with an increase in concentration of gentamicin sulfate which reduces the gelatin crosslinking by increasing amino groups in the solution

Gentamicin sulfate incorporation in the same formulation affects the crosslinking of gelatin by increasing ε-amino groups. The data represented in Figure 5 give the idea about effect of gentamicin sulfate on gelatin crosslinking. Amount of formaldehyde to be used for complete crosslinking of gelatin formulation containing gentamicin sulfate can be determined by determination of ε-amino groups present in gelatin along with gentamicin sulfate.

Evaluation of results from Figure 4 gives idea about selection of concentration of formaldehyde as a crosslinking agent for complete crosslinking of gelatin which will be safe and effective for using it as a biomaterial. Evaluation of Figure 5 indicates that an increase in concentration of gentamicin sulfate in gelatin formulations reduces the crosslinking of gelatin due to increase in amino groups. Due to the presence of higher concentration of amino groups, more amount of formaldehyde is required for crosslinking of such formulations. It was observed that gentamicin sulfate needs 6-7 mg/ml of formaldehyde for complete crosslinking.

This combined method of the TNBS assay and chromotropic acid colorimetric assay can be utilized as a major tool for quantitative determination of a crosslinking agent, formaldehyde for crosslinking of gelatin alone as well as in the presence of other amino groups.

## CONCLUSION

A procedure developed with combination of the trinitro benzenesulphonic acid (TNBS) assay with colorimetric estimation using the chromotropic acid reagent was found to be effective for determination of concentration of formaldehyde used for crosslinking of gelatin in the presence of ε-amino groups. It is likely that this procedure can be applied for determination of amount of formaldehyde as a crosslinking agent in wide range of gelatin formulations in the presence of amino groups. It also can be used for determining effective and safe concentration of formaldehyde along with other proteinaceous material.
